# Active Vision in Binocular Depth Estimation: A Top-Down Perspective

**DOI:** 10.3390/biomimetics8050445

**Published:** 2023-09-21

**Authors:** Matteo Priorelli, Giovanni Pezzulo, Ivilin Peev Stoianov

**Affiliations:** 1 Institute of Cognitive Sciences and Technologies, National Research Council of Italy, 35137 Padova, Italy; matteo.priorelli@gmail.com; 2 Institute of Cognitive Sciences and Technologies, National Research Council of Italy, 00185 Rome, Italy; giovanni.pezzulo@cnr.it

**Keywords:** active inference, depth perception, active vision, predictive coding, action-perception cycles

## Abstract

Depth estimation is an ill-posed problem; objects of different shapes or dimensions, even if at different distances, may project to the same image on the retina. Our brain uses several cues for depth estimation, including monocular cues such as motion parallax and binocular cues such as diplopia. However, it remains unclear how the computations required for depth estimation are implemented in biologically plausible ways. State-of-the-art approaches to depth estimation based on deep neural networks implicitly describe the brain as a hierarchical feature detector. Instead, in this paper we propose an alternative approach that casts depth estimation as a problem of active inference. We show that depth can be inferred by inverting a hierarchical generative model that simultaneously predicts the eyes’ projections from a 2D belief over an object. Model inversion consists of a series of biologically plausible homogeneous transformations based on Predictive Coding principles. Under the plausible assumption of a nonuniform fovea resolution, depth estimation favors an active vision strategy that fixates the object with the eyes, rendering the depth belief more accurate. This strategy is not realized by first fixating on a target and then estimating the depth; instead, it combines the two processes through action–perception cycles, with a similar mechanism of the saccades during object recognition. The proposed approach requires only local (top-down and bottom-up) message passing, which can be implemented in biologically plausible neural circuits.

## 1. Introduction

Depth estimation is a complex process involving continuous activation of every level of the visual cortex and even higher-level regions. Disparity-sensitive cells of a different kind can be found early in the visual cortex [1,2], and it seems that the resulting signals travel through the dorsal and ventral pathways for different purposes; parietal regions (in particular, the anterior and lateral intraparietal regions) make major contributions to depth estimation for visually-guided actions in hand and eye movements [3,4], while the inferotemporal cortex supports the creation of 3D shapes based on the relative disparity between objects [2,5]. The brain can rely on several cues to estimate the depth of objects, the most important ones being (i) binocular disparity, which allows the visual cortex to have access to two different perspectives of the same environment; (ii) the motion parallax effect, which happens when objects at a greater distance move slower than nearby objects; and (iii) the angular difference between the eyes when fixating on the same object (*vergence*). However, the exact contributions of these mechanisms to the overall process of depth estimation, and critically where and how the information processing of these signals occurs, remains unclear.

Traditionally, the visual cortex has been associated with a feature detector: as the sensory signals climb the hierarchy, more complex features are detected by increasing cortical levels, such that high-level representations of objects are constructed from lines and contours. This view has inspired the development of Convolutional Neural Networks, which have led to remarkable results in object recognition tasks [6]. Despite its success, this bottom-up approach is not able to capture several top-down mechanisms that affect our everyday perception of the external world, as in the case of visual illusions [7]. In recent years, a different perspective has emerged based on Predictive Coding theories that views these illusions not just as unexpected phenomena but as expressions of the main mechanism through which our brain is able to efficiently predict and act over the environment [8,9]. Under this view, the biases we perceive are actually hints to better minimize the errors between our sensations and our predictions [10]. Furthermore, vision is increasingly considered to be an active process that constantly tries to reduce the uncertainty of what will happen next.

In this article, we apply this predictive and inferential view of perception to depth estimation. Specifically, we advance an Active Inference model that is able to estimate the depth of an object based on two projected images through a process of prediction error minimization and active oculomotor behavior. In our model, depth estimation does not consist of a bottom-up process that detects disparities in the images of the two eyes, but of an inference of top-down projective predictions from a high-level representation of the object. In other words, the estimation of the object’s depth naturally arises by inverting a visual generative model wherein the resulting prediction errors flow up the cortical hierarchy, which contrasts with the direct processes occurring in neural networks.

## 2. Materials and Methods

**Figure 1:**
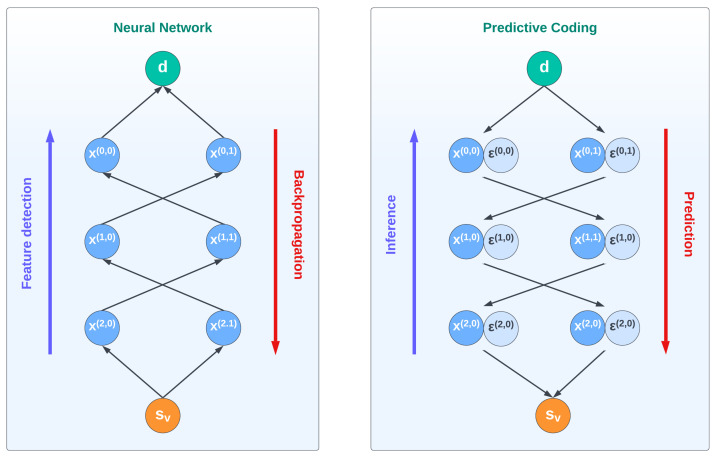
Information processing in neural networks (**left**) and Predictive Coding (**right**). In a neural network, the visual observation $s_{v}$ travels through the cortical hierarchy in a bottom-up way, detecting increasingly more complex features $x^{\left(i,j\right)}$ and eventually estimating the depth of an object d. The descending projections are considered here as feedback signals that convey backpropagation errors. In contrast, in a Predictive Coding Network the depth d is a high-level belief generating a visual prediction that is compared with the observation. This process leads to a cascade of prediction errors $\varepsilon^{\left(i,j\right)}$ associated with each intermediate prediction $x^{\left(i,j\right)}$ that are minimized throughout the hierarchy, eventually inferring the correct belief (for details, see the Section 2).

The theory of Active Inference assumes that an agent is endowed with a generative model that makes predictions over sensory observations [10,11,12,13], as shown in Figure 1. The discrepancy between predictions and observations generates a prediction error that is minimized in order to deal with a dynamical environment and to anticipate what will happen next. This generative model hinges on three components encoded in generalized coordinates of increasing temporal orders (e.g., position, velocity, acceleration, etc.): hidden states $\tilde{x}$, hidden causes $\tilde{v}$, and sensory signals $\tilde{s}$. These components are expressed through a nonlinear system that defines the prediction of sensory signals and the evolution of hidden states and causes across time:

(1) $$\begin{array}{c} \begin{array}{cc} \tilde{s} & =\tilde{g}\left(\tilde{x}\right)+w_{s}\\ D\tilde{x} & =\tilde{f}\left(\tilde{x},\tilde{v}\right)+w_{x} \end{array} \end{array}$$

In this context, D denotes a differential operator that shifts all temporal orders by one, such as $D\tilde{x}=\left[x^{\prime },x^{\prime \prime },x^{\prime \prime \prime },\ldots \right]$. Furthermore, $w_{s}$ and $w_{x}$ stand as noise terms drawn from a Gaussian distribution. The considered joint probability is divided into distinct distributions:

(2) $$p\left(\tilde{s},\tilde{x},\tilde{v}\right)=p\left(\tilde{s}|\tilde{x}\right)p\left(\tilde{x}|\tilde{v}\right)p\left(\tilde{v}\right)$$

Typically, each distribution is approximated with Gaussian functions:

(3) $$\begin{array}{c} \begin{array}{cc} p(\tilde{s}|\tilde{x}) & =N(\tilde{g}\left(\tilde{x}\right),{\tilde{\pi }}_{s}^{-1})\\ p(\tilde{x}|\tilde{v}) & =N(\tilde{f}\left(\tilde{x},\tilde{v}\right),{\tilde{\pi }}_{x}^{-1})\\ p(\tilde{v}|\eta ) & =N(\eta ,{\tilde{\pi }}_{v}^{-1}) \end{array} \end{array}$$

where η is a prior, while the distributions are expressed in terms of precisions (or inverse variances) ${\tilde{\pi }}_{s}$, ${\tilde{\pi }}_{x}$, and ${\tilde{\pi }}_{v}$.

Following a variational inference method [14], these distributions are inferred via approximate posteriors $q(\tilde{x})$ and $q(\tilde{v})$. Under appropriate assumptions, minimizing the Variational Free Energy (VFE) F, defined as the disparity between the KL divergence of real and approximate posteriors and the log evidence

(4) $$F=\underset{q(\tilde{x})}{E}\left[\ln\frac{q(\tilde{x})}{p(\tilde{x},\tilde{y})}\right]=\underset{q(\tilde{x})}{E}\left[\ln\frac{q(\tilde{x})}{p(\tilde{x}|\tilde{y})}\right]-\ln p\left(\tilde{y}\right),$$

leads to the minimization of prediction errors. The belief updates $\tilde{\mu }$ and $\tilde{\nu }$ concerning hidden states and hidden causes, respectively, expanded as follows:

(5) $$\begin{array}{c} \begin{array}{cc} \dot{\tilde{\mu }}-D\tilde{\mu } & =-\partial_{\mu }F=\partial {\tilde{g}}^{T}{\tilde{\pi }}_{s}{\tilde{\varepsilon }}_{s}+\partial_{\mu }{\tilde{f}}^{T}{\tilde{\pi }}_{\mu }{\tilde{\varepsilon }}_{\mu }-D^{T}{\tilde{\pi }}_{\mu }{\tilde{\varepsilon }}_{\mu }\\ \dot{\tilde{\nu }}-D\tilde{\nu } & =-\partial_{\nu }F=\partial_{\nu }{\tilde{f}}^{T}{\tilde{\pi }}_{\mu }{\tilde{\varepsilon }}_{\mu }-{\tilde{\pi }}_{\nu }{\tilde{\varepsilon }}_{v} \end{array} \end{array}$$

where, ${\tilde{\varepsilon }}_{s}$, ${\tilde{\varepsilon }}_{\mu }$, and ${\tilde{\varepsilon }}_{\nu }$ denote the prediction errors of the sensory signals, dynamics, and priors.

(6) $$\begin{array}{cc} {\tilde{\varepsilon }}_{s} & =\tilde{s}-\tilde{g}\left(\tilde{\mu }\right)\\ {\tilde{\varepsilon }}_{\mu } & =D\tilde{\mu }-\tilde{f}\left(\tilde{\mu },\tilde{\nu }\right)\\ {\tilde{\varepsilon }}_{\nu } & =\tilde{\nu }-\eta \end{array}$$

A simple Active Inference scheme can handle various tasks, yet the effectiveness of the theory stems from a hierarchical structure that enables the brain to grasp the hierarchical associations between sensory observations and their causes [15]. Specifically, the model delineated above can be expanded by linking each hidden cause with another generative model; as a result, the prior becomes the prediction from the layer above, while the observation becomes the likelihood of the layer below.

(7) $$\begin{array}{cc} {\tilde{\varepsilon }}_{\mu }^{\left(j\right)} & =D{\tilde{\mu }}_{\mu }^{\left(j\right)}-{\tilde{f}}^{\left(j\right)}\left({\tilde{\mu }}^{\left(j\right)},{\tilde{\nu }}^{\left(j\right)}\right)\\ {\tilde{\varepsilon }}_{\nu }^{\left(j\right)} & ={\tilde{\mu }}_{\nu }^{\left(j+1\right)}-{\tilde{g}}^{\left(j\right)}\left({\tilde{\mu }}^{\left(j\right)}\right) \end{array}$$

In contrast, the execution of action is accomplished by minimizing the proprioceptive component of the VFE concerning the motor control signals a:

(8) $$\dot{a}=-\partial_{a}F_{p}=-\partial_{a}{\tilde{s}}_{p}{\tilde{\pi }}_{p}{\tilde{\varepsilon }}_{p}$$

where $\partial_{a}s_{p}$ stands for the partial derivative of proprioceptive observations regarding the motor control signals, ${\tilde{\pi }}_{p}$ are the precisions of the proprioceptive generative model, and ${\tilde{\varepsilon }}_{p}$ are the generalized proprioceptive prediction errors:

(9) $${\tilde{\varepsilon }}_{p}={\tilde{s}}_{p}-{\tilde{g}}_{p}\left(\tilde{\mu }\right).$$

In summary, in Active Inference, goal-directed behavior is generally possible by first biasing the belief over the hidden states through a specific cause. This cause acts as a prior that encodes the agent’s belief about the state of affairs of the world. In this context, action follows because the hidden states generate a proprioceptive prediction error that is suppressed through a reflex arc [16]. For instance, supposing that the agent has to rotate the arm by a few degrees, the belief over the arm angle is subject to two opposing forces

(10) $$\dot{\mu }=\pi_{p}\varepsilon_{p}-\pi_{v}\varepsilon_{v},$$

one from above (pulling it toward its expectation) and one from below (pulling it toward what it is currently perceiving). The tradeoff between the two forces is expressed in terms of the precisions $\pi_{p}$ and $\pi_{v}$, which encode the agent’s level of confidence about the particular prediction errors. By appropriately tuning the precision parameters, it is possible to smoothly push the belief towards a desired state, eventually driving the real arm through Equation (8).

## 3. Results

### 3.1. Homogeneous Transformations as Hierarchical Active Inference

Classical Predictive Coding models are passive in the sense that the model cannot select its visual stimuli [8]. On the other hand, our Active Inference model can actively control “eyes” in order to sample those preferred stimuli that reduce prediction errors.

State-of-the-art implementations of oculomotor behavior in Active Inference rely on a latent state (or belief) over the eye angle, and attractors are usually defined directly in the polar domain [17,18]. While having interesting implications for simulating saccadic and smooth pursuit eye movements, such models do not consider the fact that eyes fixate on the target from two different perspectives. A similar limitation can be found in models of reaching, in which the 3D position of the object to be reached is directly provided as a visual observation [19,20]. Furthermore, because there is only a single level specified in polar coordinates, if one wants to fixate or reach a target defined in Cartesian coordinates, a relatively complex dynamics function has to be defined at that level.

**Figure 2:**
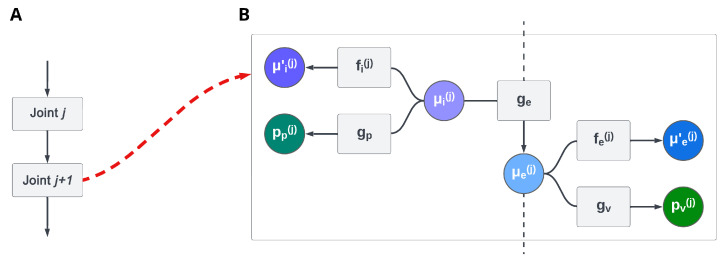
(**A**) An example of a portion of a kinematic plant. (**B**) Factor graph of a single level *j* of the hierarchical kinematic model composed of intrinsic $\mu_{i}^{\left(j\right)}$ and extrinsic $\mu_{e}^{\left(j\right)}$ beliefs. These beliefs generate proprioceptive and visual predictions $p_{p}^{\left(j\right)}$ and $p_{v}^{\left(j\right)}$ through generative models $g_{p}$ and $g_{v}$, respectively. Furthermore, the beliefs predict trajectories (here, only the velocities $\mu {{}^{\prime }}_{i}^{\left(j\right)}$ and $\mu \prime_{e}^{\left(j\right)}$) through the dynamics functions $f_{i}^{\left(j\right)}$ and $f_{e}^{\left(j\right)}$. Note that the extrinsic belief of level j−1 acts as a prior for layer *j* through a kinematic generative model $g_{e}$. See [21] for more details.

Using a *hierarchical kinematic model*—based on Active Inference—that includes both intrinsic (e.g., joint angles and limb lengths) and extrinsic (e.g., Cartesian positions) coordinates affords efficient control of a simulated body [21]. The extrinsic configuration of the motor plant is computed hierarchically, as shown in Figure 2. For the relatively simple kinematic control tasks targeted in [21], these computations only required two simple transformations between reference frames, namely, translations and rotations. However, a hierarchical kinematic model can be easily extended to more complex tasks that require different transformations.

In robotics, transformations between reference frames are usually realized through the multiplication of a linear transformation matrix. These operations can be decomposed into simpler steps where *homogeneous coordinates* are multiplied one at a time through the chain rule, allowing for more efficient computations. Specifically, if the x and y axes represent a Cartesian plane, a homogeneous representation augments the latter with an additional dimension called the *projective space*. In this new system, multiplying the point coordinates by the same factor ensures that the mapping remains unaltered, i.e., $\left(p_{x},p_{y},1\right)\equiv \left(p_{z}p_{x},p_{z}p_{y},p_{z}\right)$.

Affine transformations preserve parallel lines, and take the following form:

(11) $$T=\left[\begin{array}{ccc} t_{11} & t_{12} & t_{13}\\ t_{21} & t_{22} & t_{23}\\ 0 & 0 & 1 \end{array}\right]$$

where the last row ensures that every point always maps to the same plane. Following the chain rule, a point in the plane $p^{0}$ can be rotated and translated by multiplication of the corresponding transformations:

(12) $$p^{f}=\left[\begin{array}{c} p_{x}^{f}\\ p_{y}^{f}\\ 1 \end{array}\right]=\left[\begin{array}{ccc} c_{\theta } & -s_{\theta } & 0\\ s_{\theta } & c_{\theta } & 0\\ 0 & 0 & 1 \end{array}\right]\cdot \left[\begin{array}{ccc} 1 & 0 & l_{x}\\ 0 & 1 & l_{y}\\ 0 & 0 & 1 \end{array}\right]\cdot p^{0}=\left[\begin{array}{c} c_{\theta }\left(p_{x}^{0}+l_{x}\right)-s_{\theta }\left(p_{y}^{0}+l_{y}\right)\\ s_{\theta }\left(p_{x}^{0}+l_{x}\right)+c_{\theta }\left(p_{y}^{0}+l_{y}\right)\\ 1 \end{array}\right]$$

where $s_{\theta }$ and $c_{\theta }$ are the sine and cosine of the rotation θ and $l_{x}$ and $l_{y}$ are the coordinates of the translation.

By appropriately changing the values of the matrix, additional affine transformations such as shearing or scaling can be obtained. Critically, if the last row is modified it is possible to realize perspective projections:

(13) $$p^{f}=\left[\begin{array}{c} p_{x}^{f}\\ p_{y}^{f}\\ 1 \end{array}\right]=\left[\begin{array}{ccc} 1 & 0 & 0\\ 0 & 1 & 0\\ z_{x} & z_{y} & 1 \end{array}\right]p^{0}=\left[\begin{array}{c} p_{x}^{0}\\ p_{y}^{0}\\ z_{x}p_{x}^{0}+z_{y}p_{y}^{0}+1 \end{array}\right]$$

such that the new point is no longer mapped on the same plane $p_{z}=1$. Thus, to map it back to the Cartesian plane, we can divide the x and y coordinates by the last element:

(14) $$\left[\begin{array}{c} p_{x}^{0}\\ p_{y}^{0}\\ z_{x}p_{x}^{0}+z_{y}p_{y}^{0}+1 \end{array}\right]\equiv \left[\begin{array}{c} \frac{p_{x}^{0}}{z_{x}p_{x}^{0}+z_{y}p_{y}^{0}+1}\\ \frac{p_{y}^{0}}{z_{x}p_{x}^{0}+z_{y}p_{y}^{0}+1}\\ 1 \end{array}\right].$$

This special transformation is critical for computer vision, as it allows points to be projected on an image plane or the depth of an object to be estimated. If we have a 3D point $a=(a_{x},a_{y},a_{z},1)$ expressed in homogeneous coordinates, we can obtain the corresponding 2D point p projected in the camera plane by first performing a roto-translation similar to Equation (12) through a matrix that encodes the location and orientation of the camera (i.e., the *extrinsic parameters*):

(15) $$r=\left[\begin{array}{c} r_{x}\\ r_{y}\\ r_{z}\\ 1 \end{array}\right]=\left[\begin{array}{cc} R^{T} & -R^{T}t\\ 0^{T} & 1 \end{array}\right]w$$

and then scaling and converting the point to 2D through the so-called *camera matrix*:

(16) $$p^{\prime }=\left[\begin{array}{ccc} f & 0 & 0\\ 0 & f & 0\\ 0 & 0 & 1 \end{array}\right]r=\left[\begin{array}{c} fr_{x}\\ fr_{y}\\ r_{z} \end{array}\right].$$

**Figure 3:**
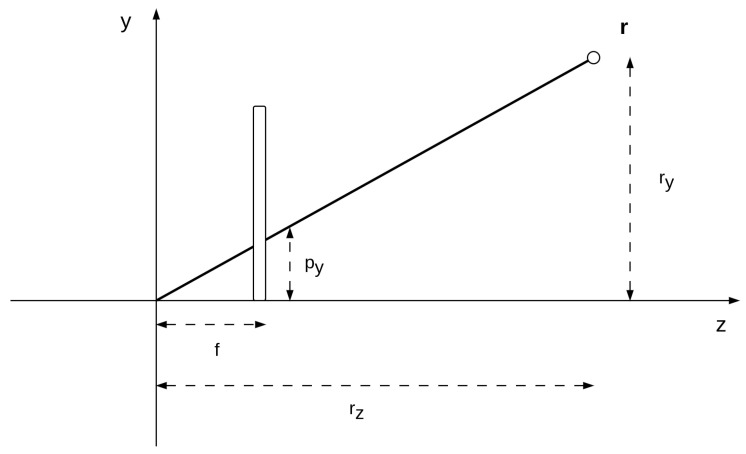
Projection of a 3D point in the camera plane (only two dimensions are shown). The y coordinates of the real point $r_{y}$ and projected point $p_{y}$ are related by the ratio between the focal length *f* and the real point depth $r_{z}$.

The projection is then performed by multiplying the depth coordinate z by the focal length *f*, which represents the distance of the image plane from the origin. As before, because the homogeneous representation is up to a scale factor, in order to transform the point $p^{\prime }$ into the Cartesian space we can divide the camera coordinates by the depth coordinate $p_{z}^{\prime }$, as shown in Figure 3,

(17) $$\begin{array}{cccc} p_{x} & =\frac{fr_{x}}{r_{z}} & \;\;\;\;\;\;\;\;\;\;\;\;\;\;\;\;\;\;\;\;p_{y} & =\frac{fr_{y}}{r_{z}} \end{array}$$

Keeping the above in mind, we can generalize the deep kinematic model of [21] by assuming that each level sequentially applies a series of homogeneous transformations. Specifically, the first belief, called $\mu_{t}^{\left(i\right)}$, contains information about a particular transformation (e.g., by which angle to rotate or by which length to translate a point).

**Figure 4:**
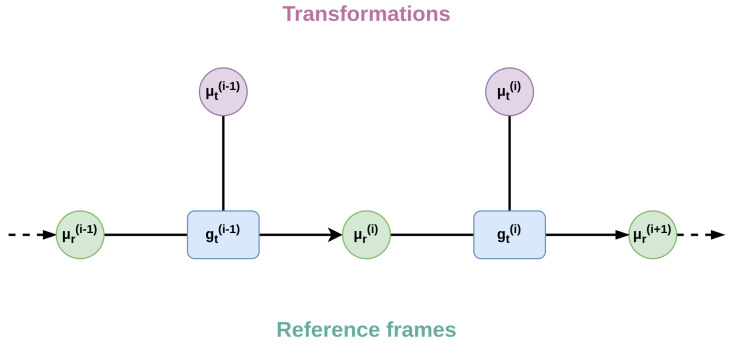
Representation of the hierarchical relationships of a generalized model with homogeneous transformations. The belief over a reference frame $\mu_{r}$ of level *i* is passed to a function $g_{t}$ encoding a homogeneous transformation along with a belief over a particular transform $\mu_{t}$ (e.g., the angle for rotation or length for translation), generating the reference frame of level i+1.

This belief then generates a homogeneous transformation relative to that Degree of Freedom (DoF), which is multiplied by a second belief expressed in a particular reference frame, as exemplified in Figure 4.

(18) $$\mu_{r}^{\left(i+1\right)}=g_{t}^{\left(i\right)}\left(\mu_{t}^{\left(i\right)},\mu_{r}^{\left(i\right)}\right)=T^{\left(i\right)}\left(\mu_{t}^{\left(i\right)}\right)\cdot \mu_{r}^{\left(i\right)}$$

The above equation leads to simple gradient computations through the generated prediction error $\varepsilon_{r}^{\left(i+1\right)}$, which is needed to iteratively update the two beliefs:

(19) $$\begin{array}{c} \begin{array}{cc} {\frac{\partial g_{t}^{\left(i\right)}}{\partial \mu_{r}^{\left(i\right)}}}^{T}\varepsilon_{r}^{\left(i+1\right)} & ={T^{\left(i\right)}}^{T}\cdot \varepsilon_{r}^{\left(i+1\right)}\\ {\frac{\partial g_{t}^{\left(i\right)}}{\partial \mu_{t}^{\left(i\right)}}}^{T}\varepsilon_{r}^{\left(i+1\right)} & =\frac{\partial T^{\left(i\right)}}{\partial \mu_{t}^{\left(i\right)}}⊙\left[\varepsilon_{r}^{\left(i+1\right)}\cdot {\mu_{r}^{\left(i\right)}}^{T}\right] \end{array} \end{array}$$

where ⊙ is the element-wise product.

### 3.2. A Hierarchical Generative Model for Binocular Depth Estimation

In this section, we explain how depth estimation arises by inverting the projective predictions of the two eyes using a hierarchical generative model. For simplicity, we consider an agent interacting with a 2D world, where the depth is the *x* coordinate. Nonetheless, the same approach could be used to estimate the depth of a 3D object. We construct the generative model hierarchically, starting from a belief $\mu_{a}=\left(\mu_{a,x},\mu_{a,y},1\right)$ about the absolute 2D position of an object encoded in homogeneous coordinates, where $\mu_{a,x}$ is the depth belief. Then, two parallel pathways generate specular predictions $p_{r}^{\left(i\right)}$ that receive the eye angles encoded in a common vergence-accommodation belief $\mu_{\theta }=\left(\theta_{a},\theta_{v}\right)$ and transform the absolute coordinates of the object into the two reference frames relative to the eyes:

(20) $$p_{r}^{\left(i\right)}=g_{a}^{\left(i\right)}\left(\mu_{a},\mu_{\theta }\right)=T^{\left(i\right)}\left(\mu_{\theta }\right)\cdot \mu_{a}$$

where $T^{\left(i\right)}\left(\mu_{\theta }\right)$ is the homogeneous transformation corresponding to the extrinsic parameters of the camera:

(21) $$T^{\left(i\right)}\left(\mu_{\theta }\right)=\left[\begin{array}{ccc} c_{\theta }^{\left(i\right)} & s_{\theta }^{\left(i\right)} & -l^{\left(i\right)}s_{\theta }^{\left(i\right)}\\ -s_{\theta }^{\left(i\right)} & c_{\theta }^{\left(i\right)} & -l^{\left(i\right)}c_{\theta }^{\left(i\right)}\\ 0 & 0 & 1 \end{array}\right]$$

where $l^{\left(i\right)}$ is the distance between an eye and the origin (i.e., the middle of the eyes) and the absolute eye angles are as shown below.

(22) $$\begin{array}{cccc} \theta^{\left(0\right)} & =\theta_{a}-\theta_{v} & \;\;\;\;\;\;\;\;\;\;\;\;\;\;\;\theta^{\left(1\right)} & =\theta_{a}+\theta_{v} \end{array}$$

Each of these beliefs generates a prediction over a point projected to the corresponding camera plane.

(23) $$p_{c}^{\left(i\right)}=g_{r}\left(\mu_{r}^{\left(i\right)}\right)=\left[\begin{array}{ccc} 1 & 0 & 0\\ 0 & f & 0 \end{array}\right]\cdot \frac{\mu_{r}^{\left(i\right)}}{\mu_{r,x}^{\left(i\right)}}$$

**Figure 5:**
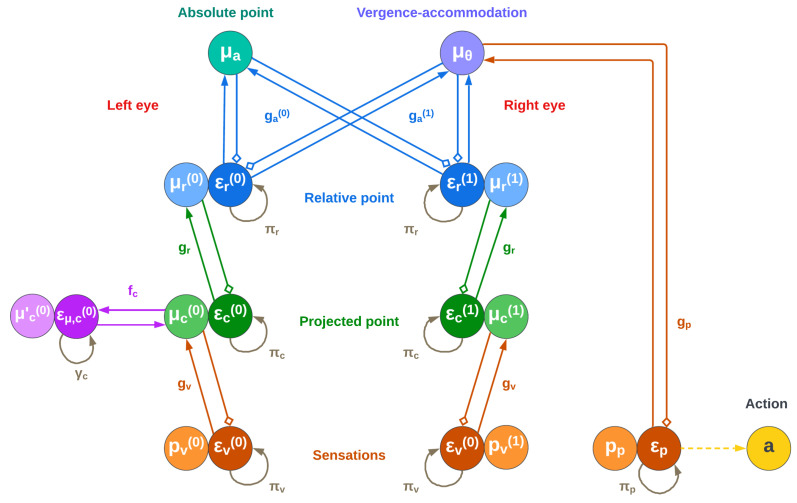
Neural-level implementation of a hierarchical generative model to estimate the depth of a point through Active Inference. The small squares indicate inhibitory connections. Unlike a neural network, depth is estimated by first generating two predictions $p_{r}^{\left(i\right)}$ of the point relative to each eye from a point in the absolute coordinates $\mu_{a}$ and vergence-accommodation angles $\mu_{\theta }$. This new belief is in turn used to compute a projection $p_{c}^{\left(i\right)}$ and finally a visual prediction $p_{v}^{\left(i\right)}$. The predictions are then compared with the visual observations, generating prediction errors throughout the hierarchy and eventually driving the beliefs at the top toward the correct values. Note that eye movements are directly triggered to suppress the proprioceptive prediction error $\varepsilon_{p}$. Intentional eye movements (e.g., for target fixation) can instead be achieved by setting a prior in the dynamics function $f_{c}$ of the belief over the projected point $\mu_{c}$ (note that for better readability the figure only shows the dynamics function $f_{c}$.

Figure 5 provides a neural-level illustration of the model, with the two branches originating from the two beliefs at the top. Note that while the eye angles belief $\mu_{\theta }$ generates separate predictions for the two eyes, proprioceptive predictions directly encode angles in the vergence-accommodation system, which is used for action execution [10].

The absolute point belief (encoded in generalized coordinates up to the second level) is updated as follows:

(24) $$\begin{array}{c} \begin{array}{cc} {\dot{\tilde{\mu }}}_{a} & =\left[\begin{array}{c} \mu_{a}^{\prime }+\sum_{i}\partial_{a}g_{a}^{T}\varepsilon_{r}^{\left(i\right)}+\partial f_{a}^{T}\varepsilon_{\mu ,a}\\ -\varepsilon_{\mu ,a} \end{array}\right]\\ {\dot{\varepsilon }}_{r}^{\left(i\right)} & =\mu_{r}^{\left(i\right)}-g_{a}^{\left(i\right)}\left(\mu_{a},\mu_{\theta }\right)-\varepsilon_{r}^{\left(i\right)}/\pi_{r}^{\left(i\right)}\\ {\dot{\varepsilon }}_{\mu ,a} & =\mu_{a}^{\prime }-f_{a}\left(\mu_{a}\right)-\varepsilon_{\mu ,a}/\gamma_{a} \end{array} \end{array}$$

where $\pi_{r}^{\left(i\right)}$ and $\varepsilon_{r}^{\left(i\right)}$ are the precisions and prediction errors of the beliefs below and $f_{a}$, $\gamma_{a}$, and $\varepsilon_{\mu ,a}$ are the function, precision, and prediction error of the dynamics of the same belief.

Thus, this belief is subject to different prediction errors $\varepsilon_{r}^{\left(i\right)}$ coming from the two eyes; the depth of the point is estimated by averaging these two pathways. Furthermore, an attractor can be defined in the dynamics function $f_{a}$ if one wishes to control the object encoded in absolute coordinates, e.g., for reaching or grasping tasks.

Similarly, the belief update equation for $\mu_{\theta }$ is as follows:

(25) $$\begin{array}{c} \begin{array}{cc} {\dot{\tilde{\mu }}}_{\theta } & =\left[\begin{array}{c} \mu_{\theta }^{\prime }+\sum_{i}\partial_{\theta }g_{a}^{T}\varepsilon_{r}^{\left(i\right)}+\partial g_{p}^{T}\varepsilon_{p}+\partial f_{\theta }^{T}\varepsilon_{\mu ,\theta }\\ -\varepsilon_{\mu ,\theta } \end{array}\right]\\ {\dot{\varepsilon }}_{p} & =s_{p}-g_{p}\left(\mu_{\theta }\right)-\varepsilon_{p}/\pi_{p}\\ {\dot{\varepsilon }}_{\mu ,\theta } & =\mu_{\theta }^{\prime }-f_{\theta }\left(\mu_{\theta }\right)-\varepsilon_{\mu ,\theta }/\gamma_{\theta } \end{array} \end{array}$$

where $\pi_{p}$, $\varepsilon_{p}$, $s_{p}$, and $g_{p}$ are the proprioceptive precision, prediction error, observation, and likelihood function (which in the following simulations is an identity mapping), while $f_{\theta }$, $\gamma_{\theta }$, and $\varepsilon_{\mu ,\theta }$ are the function, precision, and prediction error of the belief dynamics.

This belief, in addition to being affected by the proprioceptive contribution, is subject to the same prediction errors $\varepsilon_{r}^{\left(i\right)}$ in the same way as the absolute belief. In this way, the overall free energy can be minimized through two different pathways: (i) by changing the belief about the absolute location of the object (including the depth), or (ii) by modifying the angle of fixation of the eyes. As is shown in the next section, the possibility of using these pathways may create stability issues during goal-directed movements.

In such a case, an attractor can be specified in the dynamics function $f_{\theta }$ in order to explicitly control the dynamics of the eyes, e.g., by not fixating on a point on the camera plane and instead rotating the eyes along a particular direction or by a particular angle.

Finally, the belief update equation for the projected point $\mu_{c}^{\left(i\right)}$ is as follows:

(26) $$\begin{array}{c} \begin{array}{cc} {\dot{\tilde{\mu }}}_{c} & =\left[\begin{array}{c} \mu {{}^{\prime }}_{c}-\varepsilon_{c}^{\left(i\right)}+\partial g_{v}^{T}\varepsilon_{v}^{\left(i\right)}+\partial {f_{c}^{\left(i\right)}}^{T}\varepsilon_{\mu ,c}^{\left(i\right)}\\ -\varepsilon_{\mu ,c}^{\left(i\right)} \end{array}\right]\\ {\dot{\varepsilon }}_{c}^{\left(i\right)} & =\mu_{c}^{\left(i\right)}-g_{r}\left(\mu_{r}^{\left(i\right)}\right)-\varepsilon_{c}^{\left(i\right)}/\pi_{c}^{\left(i\right)}\\ {\dot{\varepsilon }}_{v}^{\left(i\right)} & =s_{v}^{\left(i\right)}-g_{v}\left(\mu_{c}^{\left(i\right)}\right)-\varepsilon_{v}^{\left(i\right)}/\pi_{v}^{\left(i\right)}\\ {\dot{\varepsilon }}_{\mu ,c}^{\left(i\right)} & =\mu_{c}^{\left(i\right)}{}^{\prime }-f_{c}^{\left(i\right)}\left(\mu_{c}^{\left(i\right)}\right)-\varepsilon_{\mu ,c}^{\left(i\right)}/\gamma_{c}^{\left(i\right)} \end{array} \end{array}$$

where $\pi_{c}^{\left(i\right)}$ and $\varepsilon_{c}^{\left(i\right)}$ are the precisions and prediction errors of the beliefs below, $\pi_{v}^{\left(i\right)}$, $\varepsilon_{v}^{\left(i\right)}$, $s_{v}^{\left(i\right)}$, and $g_{v}$ are the visual precision, prediction error, observation, and likelihood function, and $f_{c}$, $\gamma_{c}$, and $\varepsilon_{\mu ,c}$ are the function, precision, and prediction errors of the belief dynamics. Note that in the following simulations we approximate $g_{v}$ by a simple identity mapping, meaning that $s_{v}^{\left(i\right)}$ conveys a Cartesian position.

Unlike the belief over the eye angles $\mu_{\theta }$, which is only biased by the likelihoods of the levels below, this belief is subject to both a prior encoded in $\varepsilon_{c}^{\left(i\right)}$ and a visual likelihood from $\varepsilon_{v}^{\left(i\right)}$.

### 3.3. Active Vision and Target Fixation with Action–Perception Cycles

The model advanced here is not only able to infer the depth of a point, it can fixate it using active vision. This is possible by specifying an appropriate attractor in the dynamics function of the last belief $\mu_{c}^{\left(i\right)}$, or in other words, by setting an “intention” in both eyes [22] such that the projected position is at the center of the camera planes.

(27) $$\begin{array}{c} \begin{array}{cc} e_{c}^{\left(i\right)} & =\mu_{c}^{\left(i\right)}-\left(1,0\right)\\ \mu_{c}^{\left(i\right)}{}^{\prime } & =f_{c}^{\left(i\right)}\left(\mu_{c}^{\left(i\right)}\right)=\lambda e_{c}^{\left(i\right)} \end{array} \end{array}$$

In short, $f_{c}^{\left(i\right)}$ returns a velocity encoding the difference $e_{c}^{\left(i\right)}$ between the current belief and the center of the camera plane—expressed in homogeneous coordinates—multiplied by an attractor gain λ. Thus, the agent thinks that the projected point will be pulled toward the center with a velocity proportional to $e_{c}^{\left(i\right)}$. The generated prediction errors then travel back through the hierarchy, affecting both the absolute and eye angle beliefs. Because what we want in this case is to modify the latter pathway (directly generating proprioceptive predictions for movement), the former pathway can be problematic. In fact, if $\mu_{a}$ already encodes the correct depth of the object, fixation occurs very rapidly; however, if this is not the case, then the prediction errors $\varepsilon_{r}^{\left(i\right)}$ are free to flow through all the open pathways, driving the beliefs in different directions and causing the free energy minimization process to become stuck with an incorrect depth coordinate and eye angles [23].

This abnormal behavior can be avoided by decomposing the task into cyclical phases of action and perception [24]. During an action phase, the absolute belief is kept fixed, meaning that the relative prediction errors $\varepsilon_{r}^{\left(i\right)}$ can only flow towards the belief over the eye angles, which results in the eyes moving according to the depth belief. During a perception phase, action is blocked (either by setting the attractor gain or the proprioceptive precision to zero), while $\varepsilon_{r}^{\left(i\right)}$ is free to flow in any direction; this has the result of pushing the depth belief toward the correct value signaled through the sensory observations. In this way, depth estimation is achieved through multiple action and perception cycles until the overall free energy is minimized.

**Figure 6:**
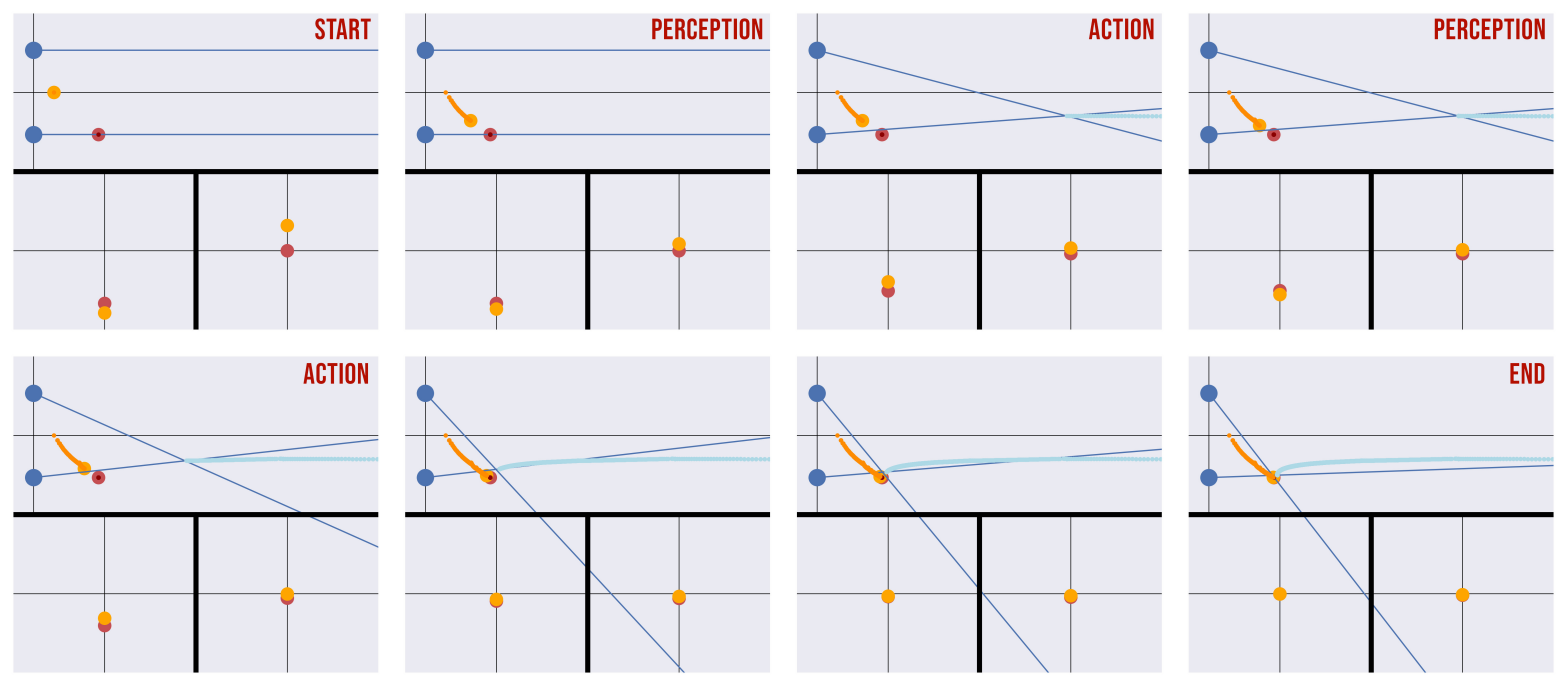
Sequence of time frames of a depth estimation task with simultaneous target fixation. The agent uses alternating action–perception phases to avoid becoming stuck during the minimization process. Each frame is composed of three images: a third-view perspective of the overall task (**top**) and a first-view perspective consisting of the projection of the target to the respective camera planes of each eye (**bottom left and bottom right**). In the top panel, the eyes are represented by blue circles and the real and estimated target positions are shown in red and orange. The fixation trajectory (when vergence occurs) is represented in cyan. The thin blue lines are the fixation angles of the eyes. In the bottom panel, the real and estimated target positions are shown in red and orange. The abscissa and ordinate respectively represent the target depth and its projection.

Figure 6 shows a sequence of time frames of a depth estimation task in which the (perceptual) process of depth estimation and the (active) process of target fixation cyclically alternate in different phases every 100 time steps. As can be seen from the visualization of the point projections, the distance between the real and estimated target positions slowly decreases while both positions approach the center of the camera planes, affording efficient depth estimation.

### 3.4. Model Comparison

We tested the model introduced in Section 3.2 and Section 3.3 in a depth estimation task that consists of inferring the 2D position of the object shown in Figure 6. We compared three different versions of the model. In the first version, the eyes are kept in a fixed position with the eyes parallel to one another (*infer parallel*). In the second and third versions, while the model can actively control the eye angles, the initial values are set at the correct target position (*infer vergence*) or at a random location (*active vision*, as displayed in Figure 6).

Furthermore, the fovea of the simulated “eye” can have either a uniform or a nonuniform resolution; in the latter condition, the object is represented with greater accuracy when it is near the point of fixation. This reflects the fact that the biological fovea has far more receptors at the center than in the peripheral vision, which has previously been modeled with an exponential link [25]. Specifically, the variability $\Sigma_{v}$ of the Gaussian error in the visual observations in our implementation (i.e., in the generative process) exponentially increases with the distance *d* between the point of fixation and the real target position:

(28) $$\Sigma_{v}=e^{d/k}$$

where *k* is a scaling factor which was equal to 1.5 in our simulations. In the uniform condition, the visual noise was set to zero.

**Figure 7:**
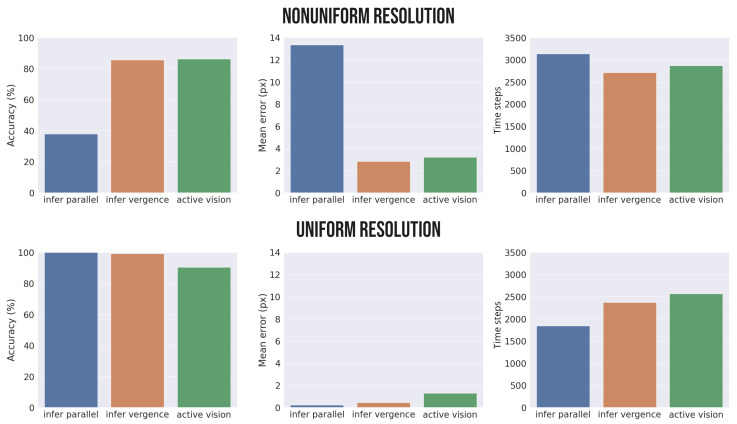
Simulation results. Performance of the depth estimation task with nonuniform (**top**) and uniform (**bottom**) foveal resolution during inference with the eyes parallel and fixed (*infer parallel*), inference with the eyes fixating on the target (*infer vergence*), and simultaneous inference and target fixation (*active vision*). The accuracy (**left panel**) measures the number of trials in which the agent successfully predicts the 2D position of the target, the mean error (**middle panel**) measures the distance between the real and estimated target positions at the end of every trial, and the time (**right panel**) measures the number of steps needed to correctly estimate the target.

Figure 7 shows the results of the simulations, including the accuracy (the number of trials in which the agent successfully predicts the 2D position of the target, left panel), mean error (distance between the real and estimated target position at the end of every trial, middle panel), and estimation time (number of steps needed to correctly estimate the target, right panel). The number of time steps for each phase was set to 100, as before. The figure shows that depth estimation with parallel eyes (*infer parallel*) in the nonuniform condition results in very low accuracy, especially when the target position is far from the fixation point. This is to be expected, as in this condition the fovea has low resolution at the periphery. If the angle of the eyes is instead set to fixate on the target (*infer vergence*), the accuracy is much higher and few fluctuations occur. Finally, the *active vision* model that simultaneously implements depth estimation and target fixation achieves a level of performance that is almost on par with the model where the fixation is initialized at the correct position. Indeed, the only appreciable difference between the last two conditions is the slightly greater number of time steps in the *active vision* condition.

This pattern of results shows three main things. First, the hierarchical Active Inference model is able to solve the depth estimation problem, as evident from its perfect accuracy in the task. Second, the model is able to infer depth as well as to simultaneously select the best way to sample its preferred stimuli, i.e., by fixating on the target. This is possible because during a trial (as exemplified graphically in Figure 6) the active vision model obtains increasingly more accurate estimates of the depth as the point of fixation approaches the target. Note that this pattern of results emerges because of the nonuniform resolution of the fovea. In fact, if the foveal resolution is assumed to be uniform (such as in camera models of artificial agents), the best accuracy is achieved by keeping the eyes parallel (Figure 7, *infer parallel* condition). In this case, fixating on the target does not help depth estimation, and indeed hinders and slows it down, which is probably caused by the increased effort that the agent needs to make in order to infer the reference frames of the eyes when they are rotated in different directions. This has the consequence of further increasing the time needed for the active vision model to estimate the depth.

Intuitively, the better performance in the uniform condition is due to the lack of noise in the visual input. Although a more realistic scenario would consider noise in this case, it is reasonable to assume that it would have a much smaller amplitude due to a uniform distribution. Considering only the nonuniform sensory distribution, the better performance in the *infer vergence* condition relative to *active vision* could be due to the fact that in the former case the agent starts the inference process from a state of fixation on the correct 3D position. Thus, an active vision strategy in the *infer vergence* condition only needs to estimate the object’s depth. In comparing these two scenarios, it can be noted that *active vision* performs almost optimally, similar to the *infer vergence* condition when the eye angles are set to the correct values for object fixation. However, the latter condition rarely occurs in a realistic setting, and a more meaningful depth estimation comparison would be between *active vision* and the more general case in which the agent is fixating another object or not fixated on anything in particular, which we approximate with the *infer parallel* simulation.

## 4. Discussion

We have advanced a hierarchical Active Inference model for depth estimation and target fixation operating in the state space of the projected camera planes. Our results show that depth estimation can be solved by inference, that is, by inverting a hierarchical generative model that predicts the projection of the eyes from a 2D belief over an object. Furthermore, our results show that active vision dynamics makes the inference particularly effective and that fixating the target drastically improves task accuracy (see Figure 7). Crucially, the proposed model can be implemented in biologically plausible neural circuits for Predictive Coding [8,9,10], which only require local (top-down and bottom-up) message passing. From a technical perspective, our model shows that inference can be iteratively realized in any homogeneous transformation by combining generative models at different levels, each of which computes a specific transformation, which could be, for instance, a roto-translation for kinematic inference [21] or a projection for computer vision.

This proposal has several elements of novelty compared to previous approaches to depth estimation. First, by focusing on inference and local message passing, our proposal departs from the trend of viewing cortical processing from a purely bottom-up perspective. The latter is common in neural network approaches, which start from the image of an object and gradually detect more and more complex features, eventually estimating its depth. Moreover, our proposal is distinct from a direct approach that generates the depth of an object from a top-down perspective, e.g., using vergence cues. The role of vergence has long been considered key in facilitating binocular fusion [5] and maximizing coding efficiency in a single environmental representation [26]; however, recent studies have dramatically reduced the importance of this mechanism in depth estimation. Binocular fusion might not be strictly necessary for this task [27], as90% performance of depth estimation is attributed to diplopia [28] and it has never actually been tested as an absolute distance cue without eliminating all possible confounders [28]. Moreover, when fixating a target there is always a disparity of vertical fixation in monocular images, with no line precisely intersecting to form a vergence angle [29]; it has been demonstrated that vergence does not correspond to the exact distance of the object being gazed at [30]. In keeping with this body of evidence, the vergence belief does not play a critical role in depth estimation in our model, only operating along with a high-level belief over the 2D position of the object in order to predict the projections for the two eyes. These projections are compared with the visual observations, and the resulting prediction errors that flow back through the hierarchy then drive the update of both beliefs (i.e., about the eye angles and the 2D object position). This change occurs in two possible ways: (i) to the estimated depth of the object or (ii) to the vergence-accommodation angles of the eyes, ultimately realizing a specific movement. In sum, depth estimation is not purely a top-down process; rather, it is realized through the inversion of a generative projective model and by averaging the information obtained through the two parallel pathways of the eyes. In conclusion, our model supports the direct (from disparity to both vergence and depth) rather than the indirect (from disparity to vergence and then to depth) hypothesis of depth estimation [27]. This account is in line with the fact that small changes in vergence (*delta theta*) are a consequence of, and not a direct hint about, depth estimation [28], as well as that reflex-like vergence mechanisms serve only to eliminate small vergence errors, not to actively transfer the gaze to new depth planes [31].

An interesting consequence of this architecture is that, in contrast to standard neural networks, it permits the imposition of priors over the depth belief in order to drive and speed up the inferential process. Such priors may come through different sensory modalities or other visual cues, e.g., motion parallax or perspective, which we have not considered here. This is in line with the finding that vergence alone is unable to predict depth with ambiguous cues [5], suggesting that depth belief is constantly influenced by top-down mechanisms and higher-level cues, and does not simply arise directly from perception. In addition to depth priors, using an Active Inference model has the advantage that, if one wishes to fixate on a target, simple attractors can be defined at either the eye angle beliefs or the last projection level, each in their own domain. For example, requiring that the agent should perceive a projection of an object at the center of the camera plane results in the generation of a prediction error that ultimately moves the eyes towards that object, emphasizing the importance of active sensing strategies to enhance inference [32,33].

However, the fact that there are two open pathways through which the prediction errors of the projections can flow, i.e., the eye angles and the absolute beliefs, may be problematic in certain cases, e.g., during simultaneous depth estimation and target fixation. It is natural to think that depth estimation follows target fixation. In fact, top-down processing to verge on a target is generally not necessary; when an image is presented to a camera, the latter might move into this projected space directly, resulting in a simpler control [34,35]. Then, depth can be computed directly from vergence cues. However, our model assumes that the eye angles generate the projections by first performing a roto-translation in the 2D space using the estimated depth, allowing further mechanisms for more efficient inference. Under this assumption, target fixation in the projected space is possible through a top-down process that is constantly biased by the high-level belief. Nonetheless, a direct vergence control (not considered here) could be implemented by additional connections between the belief over the 2D or projected points and the angle beliefs.

With these considerations, it would appear plausible that the two processes of depth estimation and target fixation might run in parallel. However, when this is the case the prediction errors of the projections drive the two high-level beliefs independently towards a direction that minimizes the free energy, leading the agent to become stuck in an intermediate configuration with incorrect object depth and eye angles. One way to solve this issue that we have pursued here consists of decomposing the task into cyclical phases of action and perception [23,24]. During an action phase, the 2D belief is fixed and the agent can fixate on the predicted projections, while during a perception phase the agent can infer the 2D position of an object but is not allowed to move its sight. This implies that the prediction errors of the projections alternately flow in different directions (2D position and eye angles) one step at a time, which results in (i) the object being pulled toward the center of the camera planes and (ii) the estimated 2D position converging toward the correct one, as shown in Figure 6. Action–perception cycles have been studied in discrete time models of Active Inference; for example, cycles of saccades and visual sampling allow an agent to reduce the uncertainty over the environment, e.g., by rapidly oscillating between different points for recognizing an object [36,37]. Here, we show that action–perception cycles are useful in continuous time models, such as the one used here, to ensure effective minimization of the free energy as well as when an agent is required to reach an object with the end-effector while inferring the lengths of its limbs [23]. In summary, the two processes of recognizing a face and estimating an object’s depth can both be viewed as a process of actively accumulating sensory evidence at different timescales. From a brain-centric perspective, action–perception cycles have often been associated with hippocampal theta rhythms and cortical oscillations, which might indicate segmentation of continuous experiences into discrete elements [24,38]. From a more technical perspective, the cyclical scheme that we propose for action–perception cycles, which consists of keeping one aspect of the optimization objective fixed when updating the other, is commonly used in various optimization algorithms such as expectation maximization [39]; a similar approach is used for learning and inference in Predictive Coding Networks [40,41].

Our results show that active vision improves depth estimation. However, if vergence does not provide a useful cue for depth, then how is this possible? The answer lies in the nonuniform resolution of the fovea, which has far more receptors at the center of fixation than in the peripheral vision. It is supposed that this nonlinear resolution allows sensory processing resources to be gathered around the most relevant sources of information [42]. Under this assumption, the best performance is achieved when both eyes are fixated on the object, as shown in Figure 7. As noted in [43], when stereo cameras have a nonsymmetrical vergence angle, the error is at a minimum when the projections of a point fall at the center of the camera planes. Hence, vergence can effectively play a key role in depth estimation while providing a unified representation of the environment. This can be appreciated by considering that the *infer vergence* and *active vision* models are more accurate than the *infer parallel* model in the nonuniform resolution condition. With a uniform resolution, the error is larger when the eyes converge to the target, because the focal angle of the pixels in the center is larger than in the periphery [43]. In addition to the increased error, the estimation seems to be further slowed down by the inference of the different reference frames due to vergence. Taken together, the consequence is that in the uniform resolution scenario the best estimation is achieved with fixed parallel eyes (see Figure 7), while active vision does not bring any advantage to the task. Because maintaining parallel eyes in the uniform condition results in a slightly higher accuracy, such simulations may be useful in understanding those cases in which verging on a target would improve model performance. This might be helpful for future studies in bio-inspired robotics, especially when extending the proposed model to implement high-level mechanisms, such as higher-level priors that result from the integration of cues from different sensory modalities or attentional mechanisms that unify the visual sensations into a single experience.

The model presented in this study has a number limitations that could be addressed in future studies. Notably, we used a fixed focal length *f* during all the simulations. In a more realistic setting, the focal length might be considered as another DoF of the agent, and might be changed through the suppression of proprioceptive prediction errors in order to speed up the inferential process for objects at different distances. Furthermore, although the presented simulations only estimate the depth of a 2D point, it could potentially be extended to deal with 3D objects and account for vertical binocular disparity [44]. This would involve augmenting all the latent states with the new dimension and performing a sequence of two rotations as intermediate levels before the eye projections are predicted. Then, the vergence-accommodation belief would be extended with a new DoF, allowing the agent to fixate on 3D objects. In addition, future studies might investigate how the scaling factor in Equation (28) of the nonuniform resolution could, along with more realistic nonuniform transformations, affect performance and help to model human data (e.g., [25]). It could be useful to adopt an off-center fovea on one of the two retinas and analyze the agent’s behavior to then bring the two foveas onto the target. Finally, another interesting direction for future research would be to combine the architecture proposed here with a more sophisticated Active Inference kinematics model [21], for example embodying a humanoid robot with multiple DoFs [45,46,47,48,49]. In contrast to state-of-the-art models that provide the agent either with the 3D environment directly as a visual observation [20] or with a latent space reconstructed from a Variational Autoencoder [50], this would allow the 3D position of an object to be inferred through the projections of the eyes, then used for subsequent tasks such as reaching and grasping.

## Data Availability

Simulation details and data can be found at: github.com/priorelli/active-vision.

## References

[1] Qian N. (1997). Binocular disparity and the perception of depth. Neuron.

[2] Parker A.J. (2007). Binocular depth perception and the cerebral cortex. Nat. Rev. Neurosci..

[3] Durand J.B., Nelissen K., Joly O., Wardak C., Todd J.T., Norman J.F., Janssen P., Vanduffel W., Orban G.A. (2007). Anterior Regions of Monkey Parietal Cortex Process Visual 3D Shape. Neuron.

[4] Welchman A.E., Deubelius A., Conrad V., Bülthoff H.H., Kourtzi Z. (2005). 3D shape perception from combined depth cues in human visual cortex. Nat. Neurosci..

[5] Wismeijer D.A., Van Ee R., Erkelens C.J. (2008). Depth cues, rather than perceived depth, govern vergence. Exp. Brain Res..

[6] Isomura T., Parr T., Friston K. (2019). Bayesian filtering with multiple internal models: Toward a theory of social intelligence. Neural Comput..

[7] Gregory R.L. (1968). Perceptual illusions and brain models. Proc. R. Soc. Lond. Ser. Biol. Sci..

[8] Rao R.P., Ballard D.H. (1999). Predictive coding in the visual cortex: A functional interpretation of some extra-classical receptive-field effects. Nat. Neurosci..

[9] Friston K.J. (2005). A theory of cortical responses. Philos. Trans. R. Soc. Lond. Ser. B Biol. Sci..

[10] Parr T., Pezzulo G., Friston K.J. (2022). Active Inference: The Free Energy Principle in Mind, Brain, and Behavior.

[11] Friston K., FitzGerald T., Rigoli F., Schwartenbeck P., Pezzulo G. (2017). Active inference: A process theory. Neural Comput..

[12] Pezzulo G., Rigoli F., Friston K.J. (2018). Hierarchical active inference: A theory of motivated control. Trends Cogn. Sci..

[13] Pezzulo G., Rigoli F., Friston K. (2015). Active inference, homeostatic regulation and adaptive behavioural control. Prog. Neurobiol..

[14] Friston K., Mattout J., Trujillo-Barreto N., Ashburner J., Penny W. (2007). Variational free energy and the Laplace approximation. NeuroImage.

[15] Friston K.J., Parr T., de Vries B. (2017). The graphical brain: Belief propagation and active inference. Netw. Neurosci..

[16] Adams R.A., Shipp S., Friston K.J. (2013). Predictions not commands: Active inference in the motor system. Brain Struct. Funct..

[17] Parr T., Friston K.J. (2018). Active inference and the anatomy of oculomotion. Neuropsychologia.

[18] Adams R.A., Aponte E., Marshall L., Friston K.J. (2015). Active inference and oculomotor pursuit: The dynamic causal modelling of eye movements. J. Neurosci. Methods.

[19] Lanillos P., Cheng G. Adaptive Robot Body Learning and Estimation Through Predictive Coding. Proceedings of the 2018 IEEE/RSJ International Conference on Intelligent Robots and Systems (IROS).

[20] Pio-Lopez L., Nizard A., Friston K., Pezzulo G. (2016). Active inference and robot control: A case study. J. R. Soc. Interface.

[21] Priorelli M., Pezzulo G., Stoianov I.P. (2023). Deep kinematic inference affords efficient and scalable control of bodily movements. bioRxiv.

[22] Priorelli M., Stoianov I.P. (2023). Flexible Intentions: An Active Inference Theory. Front. Comput. Neurosci..

[23] Priorelli M., Stoianov I.P. Efficient motor learning through action-perception cycles in deep kinematic inference. Proceedings of the 4th International Workshop on Active Inference.

[24] VanRullen R. (2016). Perceptual cycles. Trends Cogn. Sci..

[25] Elsner A.E., Chui T.Y., Feng L., Song H.X., Papay J.A., Burns S.A. (2017). Distribution differences of macular cones measured by AOSLO: Variation in slope from fovea to periphery more pronounced than differences in total cones. Vis. Res..

[26] Zhu Q., Triesch J., Shi B.E. Integration of Vergence, Cyclovergence, and Saccades through Active Efficient Coding. Proceedings of the ICDL-EpiRob 2020—10th IEEE International Conference on Development and Learning and Epigenetic Robotics.

[27] Lugtigheid A.J., Wilcox L.M., Allison R.S., Howard I.P. (2013). Vergence eye movements are not essential for stereoscopic depth. Proc. R. Soc. Biol. Sci..

[28] Linton P. (2020). Does vision extract absolute distance from vergence?. Atten. Percept. Psychophys..

[29] Logvinenko A.D., Epelboim J., Steinman R.M. (2002). The role of vergence in the perception of distance: A fair test of bishop Berkeley’s claim. Spat. Vis..

[30] Jaschinski W. (1997). Fixation disparity and accommodation as a function of viewing distance and prism load. Ophthalmic Physiol. Opt..

[31] Masson G.S., Busettini C., Miles F.A. (1997). Vergence eye movements in response to binocular disparity without depth perception. Nature.

[32] Mannella F., Maggiore F., Baltieri M., Pezzulo G. (2021). Active inference through whiskers. Neural Netw..

[33] Parr T., Friston K.J. (2019). Attention or salience?. Curr. Opin. Psychol..

[34] Manzotti R., Gasteratos A., Metta G., Sandini G. (2001). Disparity Estimation on Log-Polar Images and Vergence Control. Comput. Vis. Image Underst..

[35] Gibaldi A., Vanegas M., Canessa A., Sabatini S.P. (2017). A Portable Bio-Inspired Architecture for Efficient Robotic Vergence Control. Int. J. Comput. Vis..

[36] Friston K., Adams R.A., Perrinet L., Breakspear M. (2012). Perceptions as hypotheses: Saccades as experiments. Front. Psychol..

[37] Anil Meera A., Novicky F., Parr T., Friston K., Lanillos P., Sajid N. (2022). Reclaiming saliency: Rhythmic precision-modulated action and perception. Front. Neurorobot..

[38] Lisman J.E., Jensen O. (2013). The theta-gamma neural code. Neuron.

[39] Dempster A.P., Laird N.M., Rubin D.B. (1977). Maximum likelihood from incomplete data via the EM algorithm. J. R. Stat. Soc. Ser. B (Methodol.).

[40] Whittington J.C., Bogacz R. (2019). Theories of Error Back-Propagation in the Brain. Trends Cogn. Sci..

[41] Millidge B., Tschantz A., Buckley C.L. (2022). Predictive Coding Approximates Backprop Along Arbitrary Computation Graphs. Neural Comput..

[42] Borji A., Itti L. (2013). State-of-the-art in visual attention modeling. IEEE Trans. Pattern Anal. Mach. Intell..

[43] Sahabi H., Basu A. (1996). Analysis of Error in Depth Perception with Vergence and Spatially Varying Sensing. Comput. Vis. Image Underst..

[44] Read J.C.A., Phillipson G.P., Glennerster A. (2009). Latitude and longitude vertical disparities. J. Vis..

[45] Lanillos P., Cheng G. (2020). Robot self/other distinction: Active inference meets neural networks learning in a mirror. arXiv.

[46] Ahmadi A., Tani J. (2019). A novel predictive-coding-inspired variational RNN model for online prediction and recognition. Neural Comput..

[47] Taniguchi T., Murata S., Suzuki M., Ognibene D., Lanillos P., Ugur E., Jamone L., Nakamura T., Ciria A., Lara B. (2023). World models and predictive coding for cognitive and developmental robotics: Frontiers and challenges. Adv. Robot..

[48] Çatal O., Verbelen T., Van de Maele T., Dhoedt B., Safron A. (2021). Robot navigation as hierarchical active inference. Neural Netw..

[49] De Coninck E., Verbelen T., Van Molle P., Simoens P., Dhoedt B. (2020). Learning robots to grasp by demonstration. Robot. Auton. Syst..

[50] Rood T., van Gerven M., Lanillos P. (2020). A Deep Active Inference Model of the Rubber-Hand Illusion. Proceedings of the Active Inference: First International Workshop, IWAI 2020, Co-located with ECML/PKDD 2020.

